# Abnormal repolarisation after a game of Jass

**DOI:** 10.1007/s12471-023-01843-7

**Published:** 2024-01-16

**Authors:** Hilde E. Groot, Jan A. Krikken

**Affiliations:** Department of Cardiology, University Medical Centre Groningen, University of Groningen, Hanzeplein 1, 9713 GZ Groningen, The Netherlands

An 86-year-old male patient with a medical history of atrial fibrillation and non-obstructive coronary artery disease experienced chest pain after a game of Jass. Use of sublingual nitroglycerin spray had some effect, but the chest pain persisted. Therefore, the patient’s general practitioner called an ambulance to transport the patient to the emergency department for cardiac analysis. When the ambulance arrived at the scene an electrocardiogram was made (Fig. 1). The cardiologist on call was consulted for activating the protocol for ST-elevation myocardial infarction.

**Fig. 1:**
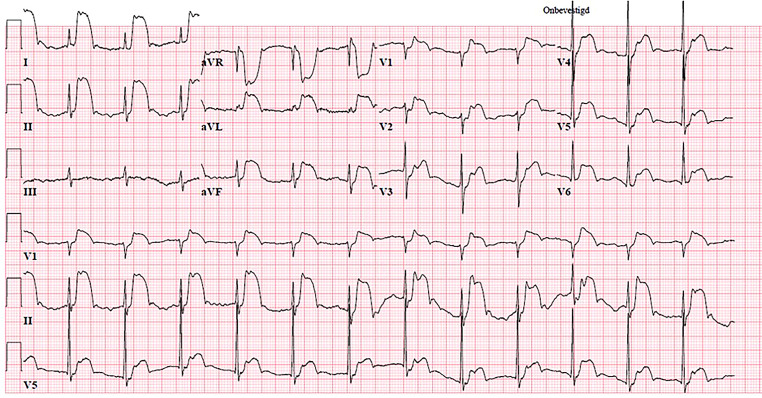
The patient’s electrocardiogram upon arrival of the ambulance

What’s the diagnosis?

## Answer

You will find the answer elsewhere in this issue.

